# Clinical analysis of a patient simultaneously positive for antibodies of myelin oligodendrocyte glycoprotein and anti–N-methyl-D-aspartate receptor: A case report

**DOI:** 10.1097/MD.0000000000024234

**Published:** 2021-01-08

**Authors:** Liming Cao, Lijie Ren, Xuming Huang

**Affiliations:** a Department of Neurology, The 3rd Affiliated Hospital of Shenzhen University; b Department of Neurology, Shenzhen University First Affiliated Hospital; c Department of Neurology, Shenzhen Second People's Hospital; d Department of Gastroenterology, Shenzhen Shiyan People's Hospital, Shenzhen, China.

**Keywords:** glycoproteins, immunotherapy, N-Methyl-D-Aspartate receptors, oligodendroglia, optic neuritis

## Abstract

**Rationale:**

Myelin oligodendrocyte glycoprotein (MOG) antibody (MOG-Ab) disease (MOG-AD) is a type of demyelinating disease of the central nervous system characterized by a high frequency of optic neuritis (ON) attacks. anti-Nmethyl-D-aspartate receptor (NMDAR) encephalitis (anti-NMDARe) is an autoimmune disorder characterized by memory deficits, conscious disturbance, and seizures. Cases of simultaneous occurrence of MOG-Ab and anti-NMDARe antibody (anti-NMDARe-Ab) are rarely reported and could be mistaken for overlapping MOG-antibody disease (MOG-AD) and NMDARe. The diagnosis of such patients is challenging.

**Patient concerns:**

We report the case of a 37-year-old man who presented with recurrent headaches for 3 months and worsening symptoms over 2 weeks. He had a history of ON. He had a generalized seizure after 7 days in the hospital.

**Diagnosis:**

Brain magnetic resonance imaging (MRI) and cerebrospinal fluid tests showed no apparent abnormalities. Repeat MRI showed slight lesions 7 days later, and cerebrospinal fluid tests showed the simultaneous occurrence of MOG-Ab and anti-NMDARe-Ab.

**Interventions:**

He completely recovered after treatment with low doses of oral corticosteroids.

**Outcomes:**

Two months and 2 years follow-up showed that his condition was stable.

**Lessons:**

The co-occurrence of MOG-Ab and anti-NMDAR-Ab does not indicate the co-occurrence of MOG-AD and anti-NMDARe. Laboratory findings should be combined with the clinical features to achieve an accurate and suitable diagnosis.

## 1 Introduction

Cases of simultaneous occurrence of myelin oligodendrocyte glycoprotein antibody (MOG-Ab) and anti-N-methyl-D-aspartate receptor encephalitis (anti-NMDARe) antibody (anti-NMDARe-Ab) are rarely reported and could be mistaken for overlapping MOG-antibody disease (MOG-AD) and NMDARe (one of the antibodies may just be a bystander). The diagnosis of such patients is challenging. MOG-Ab disease (MOG-AD) is a type of demyelinating disease of the central nervous system characterized by a high frequency of optic neuritis (ON) attacks. Anti-NMDARe is an autoimmune disorder characterized by memory deficits, conscious disturbance, and seizures.^[1–3]^ MOG-AD cases are rare, and the incidence of anti-NMDARe is 2.2/million individuals/year.^[4]^ Coexistence of MOG-Ab and anti-NMDAR-Ab in the same patient may contribute to the diversity of autoimmune diseases^[5]^; MOG antibodies have been reported in 3 of 50 patients with anti-NMDAR.^[1]^ Compared with neuromyelitis optica spectrum disorder, MOG-AD may co-exist more frequently with anti-NMDARe.^[6]^ A case of simultaneous occurrence of MOG-Ab and anti-NMDARe-Ab is a rare and unusual clinical scenario. Herein, we present such a case in a patient, wherein successful management was challenging and significant.

## 2 Case presentation

**Figure 1:**
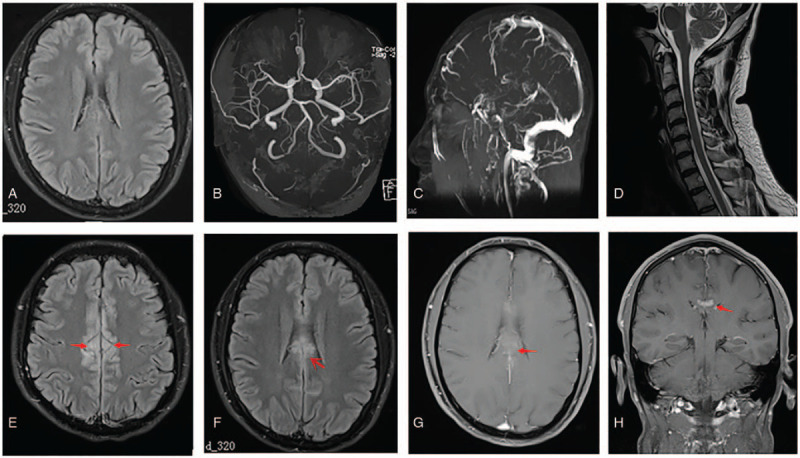
Magnetic resonance (MR) images of the brain and cervical spine. An initial fluid-attenuated inversion recovery (FLAIR) image (A), brain MR angiography (B), brain MR venography (C), and cervical spine MR images (D). All show no obvious abnormalities. Repeated FLAIR image after the condition worsened, demonstrating an abnormal hyperintense signal in the medial aspect of the bilateral frontal lobes and involvement of the cingulate cortex (E and F, arrows); a gadolinium-enhanced T1-weighted image showing enhancement in the parts above the lesions (G, arrow); and abnormal enhanced meninges (H, arrow).

A 37-year-old man was admitted in October 2018 with recurrent headaches over 3 months. His symptoms, including durative headache and left limb weakness, had worsened over the past 2 weeks. He had a history of left eye ON in 1998 and right eye ON in 2008. Both were treated successfully with intravenous dexamethasone 10 mg/day for 3 days. He had smoked 10 cigarettes daily for 20 years and denied any history of infectious diseases, alcoholism, drug abuse, and familial disease. On arrival, his temperature was 36.5°C, pulse was 93 beats/min, and blood pressure was 114/77 mm Hg. The patient was cognitively intact, and no slurred speech was observed. Examination of the cranial nerves and meningeal signs showed normal results, and his muscular strength was grade 4/5 at the distal part of the left limb. Further neurological examination did not reveal abnormalities in muscle tone and tendon jerks. Motor coordination, sensory testing, and pyramidal signs were normal. Brain and chest emergent computed tomography findings were normal. White blood cell count, platelet count, and neutrophil percentage were normal. Levels of total bilirubin, fasting blood glucose, urea, glycosylated hemoglobin, thyrotropin, C-reactive protein, and lactate dehydrogenase were within normal limits. Total cholesterol (7.03 mmol/L) and low-density lipoprotein (4.20 mmol/L) levels were increased. Tests for human immunodeficiency virus, syphilis, antineutrophil cytoplasmic, anticardiolipin, anti-myeloperoxidase, anti-proteinase 3, and thyroid antibodies, as well as an antinuclear antibody spectrum, showed negative results. No obvious abnormalities were found on an electrocardiogram, lower limb deep vein ultrasound scan, or echocardiogram. Brain magnetic resonance imaging (MRI), including T1/T2-weighted imaging, diffusion-weighted imaging, and fluid-attenuated inversion recovery imaging (Fig. 1A), showed no abnormal findings. Brain magnetic resonance angiography (Fig. 1B), brain MR venography (Fig. 1C), and cervical spine MRI (Fig. 1D) also showed that there were no obvious abnormalities. Initial cerebrospinal fluid (CSF) examination showed elevated levels of leukocytes (11 × 10^6^/L, 91% mononuclear) and proteins (482 mg/L). We initially suspected viral encephalitis. The patient was treated with intravenous *Ginkgo biloba* extract (Dr Willmar Schwabe, Karlsruhe, Germany) to improve cerebral circulation, oral alprazolam to improve sleep disorder, and traditional acupuncture. Furthermore, symptomatic treatment was also given. His sleep and headache slightly improved, but his left limb weakness persisted after the first week.

**Figure 2:**
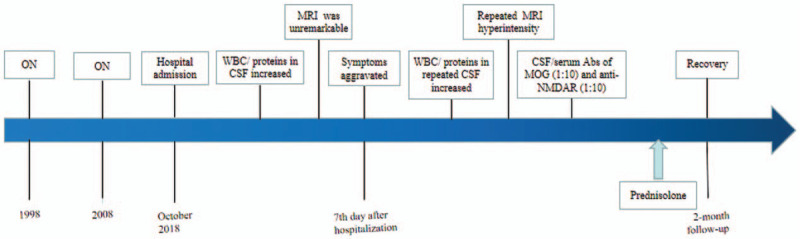
(Graphical abstract) Timeline of the patient's disease course. Abs = antibodies, Anti-NMDAR = anti-N-methyl-D-aspartate receptor, CSF = cerebrospinal fluid, MRI = magnetic resonance imaging, ON = optic neuritis, WBC = white blood cells.

On the seventh day after hospitalization, the patient had a generalized tonic–clonic seizure, which lasted for 1 to 2 min. Intramuscular injection of phenobarbital was administered to prevent recurrent seizures. No epilepsy waves were found on the emergent 24-h ambulatory electroencephalogram. Repeat CSF examination showed elevated leukocyte counts (50 × 10^6^/L, 86% mononuclear) and protein levels. No tumor cells, acid-fast bacilli, or cryptococcus were observed in a cytologic examination of the CSF. Oligoclonal bands were negative. The post-seizure brain MRI showed abnormal hyperintensity in the medial aspect of the bilateral frontal lobes (Fig. 1E and F), and enhancement of parts of the lesions (Fig. 1G) and adjacent meninges (Fig. 1H). On day 13 after admission, CSF/serum antibodies of MOG (1:10) and anti-NMDAR (1:10) were detected using a cell-based assay (Guangdong Jinyu Inspection Co, Ltd). The following CSF and serum parameters were negative: anti-contactin-associated protein-like *2*, anti-leucine-rich glioma-inactivated 1 protein, and anti-γ-aminobutyric acid receptor antibodies as well as glial cell water channel aquaporin-4 and myelin basic protein. Based on these findings, intravenous immunoglobulin therapy was recommended. However, the patient refused intravenous immunoglobulin therapy, left our hospital, and went to Hong Kong Baptist Hospital for further treatment. Repeat brain MRI (image not available) in Hong Kong showed that demyelinating disease or vasculitis could not be excluded. He was administered oral prednisolone with a gradually reducing dosage (30 mg/day for 21 days, 20 mg/day for 7 days, and 10 mg/day for 7 days). During treatment, his limb weakness and headache were completely resolved. He felt no discomfort at the 2-month follow-up (Fig. 2). We followed up with him on telephone 2 years after discharge, and his condition is stable. He was satisfied with the treatment he received and his recovery.

This study was approved by the Ethics Review Board of the 3rd Affiliated Hospital of Shenzhen University (No. 2020SZLH-LW-003). The patient provided written informed consent for participation and publication.

## 3 Discussion

Our case met the diagnostic criteria of MOG-AD,^[7]^ but not of the typical anti-NMDARe.^[8]^ The clinical findings were inconsistent with the diagnosis of overlapping MOG-AD and NMDARe.

True overlapping MOG-AD and NMDARe has been rarely reported: only one case has been reported previously.^[9]^ Therefore, patients should be carefully evaluated for MOG-AD and anti-NMDARe according to the clinical characteristics, MRI findings, and treatment effects.

**Figure 3:**
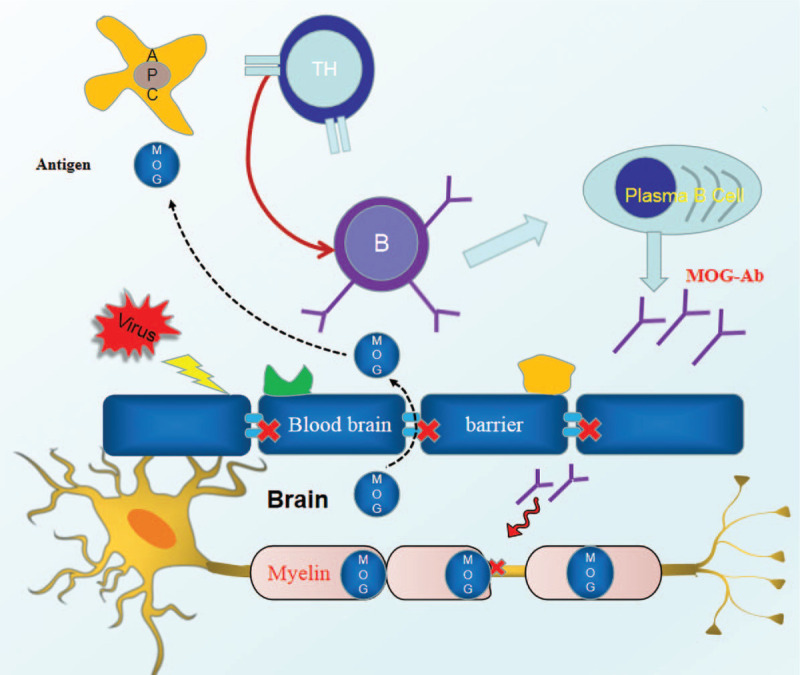
The hypothesized mechanism of action of an autoimmune reaction to MOG-AD. The MOG-antigen leaks into the peripheral blood when the blood–brain barrier is destroyed by a neurotropic viral infection. T helper cells are activated, which increases the recruitment and activation of specific B cells for MOG, which, in turn, produces a large number of MOG-Ab, causing injury to MOG in the myelin sheath and, ultimately, leading to MOG-AD. AD = antibody disease, MOG = myelin oligodendrocyte glycoprotein. Note: This figure is our own.

The hypothesized mechanism of action of an autoimmune reaction to MOG-AD is shown in Figure 3.

Most reports of patients with co-occurrence of MOG-Ab and anti-NMDAR-Ab have shown that anti-NMDARe may develop initially, followed by MOG-AD^[10]^; however, patients with the opposite occurrence^[11]^ or repetitive onset of anti-NMDARe attacks with no MOG-AD attacks (positive MOG-Ab just being a bystander) have also been reported. When a patient is simultaneously positive for MOG-Ab and anti-NMDARe-Ab, the clinical presentation can be either sequential anti-NMDARe and MOG-AD attacks (or vice versa),^[10]^ single attack,^[12–14]^ or a mixed attack.^[9]^

Specific clinical features of patients with coexistence of antibodies for MOG and anti-NMDAR are outlined in Table 1. Epilepsy is a commonly seen clinical symptom of anti-NMDARe. This patient had a seizure and did not have ON during this attack; thus, we initially suspected anti-NMDARe, despite the mild symptoms, sensitivity to steroids, and a good prognosis. MOG-AD involving the cerebral cortex can initially manifest as headache and seizure.^[15,16]^ Hence, his illness can be explained by a MOG-AD attack. Unique features of MOG-AD are the high frequency of ON attacks, good long-term neurological recovery, and sensitivity to steroid therapy.^[7]^ There is a clear distinction in the clinical characteristics (including main symptoms, MRI findings, and response to steroid treatment) between MOG-AD and anti-NMDARe. Diagnosis of the attack in terms of its association with the two diseases plays an important role in the treatment decision and prognostication. MOG-Abs is detected in patients with anti-NMDARe involving the optic nerve or other features that are inconsistent with anti-NMDARe. Similarly, when patients with MOG-AD develop atypical symptoms, such as psychosis, seizures, and dyskinesia, coexistence of anti-NMDARe should be considered.^[1,10]^ This case highlighted the importance of comprehensive screening for autoantibodies to identify unusual autoimmune diseases.

**Table 1:** Clinical features of patients with coexistence of antibodies of MOG and anti-NMDAR.

	Pérez, CA. 2020^[9]^	Zhou, L. 2017^[10]^	Aoe, S. 2019^[12]^	Zhou, J. 2018^[13]^	Sarigecili, E. 2019^[14]^
Age/sex	29-year-old/male	31-year-old/male	36-year-old/female	54-year-old/male	6-year old/male
Primary symptoms	Headache, fever, seizure, blurry vision, urinary retention, gait instability, and slurred speech in the 1st episode; bilateral painless blurry vision in the 2nd episode	Headache, seizure, fever, and headache in the 1st and the 2nd onset, respectively; right side anesthesia and continuous hiccup in the 3rd onset; blurred vision in the 4th onset	Headache, epilepsy, ataxia, depression, delusions, fever,somnolence, dyskinesia in the 1st onset; hallucinations, restlessness, tachycardia, hyperhidrosis, central apnea in the 2nd onset	Progressive dizziness, unsteadiness, narcoleptic attacks; light-headedness and intermittent spinning sensations	Alteration of gait, speech, behavior (agitation, aggression, irritability), and insomnia
Location of lesions in MRI	Lesions in mesial temporal lobe in the 1st onset; lesions in the left thalamus, optic chiasm, optic nerve, and the optic tracts in the 2nd onset	Lesions in the right temporal, parietal, and occipital cortex	Lesions in the medial aspect of the bilateral frontal lobes in the 1st onset; lesions in the basal ganglia, temporal lobe, cerebellum in the 2nd onset	Multiple lesions involvingthe bilateral cerebellum, right cerebral peduncle, and the brain parenchyma surrounding the third ventricle	Brain and spinal MRI findings were normal
Results of CSF	A mildly elevated level of protein, an elevated white blood cell count	Elevated leucocytes (80% mononuclear) and elevated protein	Protein level is unknown, mildly elevated leucocytes in the 1st and the 2nd onset	Normal opening pressure, with mild leukocytosis and elevated protein levels	No cells, normal protein and glucose levels
Cancer	No	No mention	No	Unremarkable	No mention
EEG	Unremarkable	Epileptic discharges exist	No mention	No abnormalities	Intermittent spike waves
Antibody detection	CSF anti-NMDAR-Ab (1:16); serum MOG-Ab (1:1000)	Serum anti-MOG (1:320); CSF NMDAR-Ab (1:1); OB (–)	CSF NMDAR-Ab (+) in the 1st and the 2nd episode (1:20); serum MOG-Ab (+) in the two episodes; OB (+)	CSF NMDAR-Ab (1:32); serum MOG-IgG (1:320);IgG index was 0.62; OB (–).	CSF anti-NMDAR-Ab and serum anti-MOG-Ab (1:320) all (+); OB (+); an elevated IgG index
Immunotherapy	Oral corticosteroids were initially used, and then1 course of IVMP (1 g/d × 5 days) in the 1st onset; a 5-day course of PE in the 2nd episode	IV DSM (10 mg/d) in the 1st onset; MP (80 mg/d × 2 weeks) in the 2nd onset.	3 courses of IVMP (1 course: 1 g/d × 3 days) and 1 course (0.4 g/kg × 5 days) of IVIg in the 1st onset; 2 courses of MP and PE, 1 course of IVIg in the 2nd onset.	IVMP (0.5 g/d × 3 days), and IVIG (0.4 g/kg/d × 5 days), followed by IV rituximab (0.6 g/week × 3 weeks).	MP (30 mg/kg × 3 days), followed by (20 mg/kg × 2 days).
Treatmentoutcomes	The symptoms improved after IVMP in the 1st onset. Neurological exam was normal at follow-up in the 2nd onset	The symptoms relieved after IV DSM in the 1st onset. His symptoms relieved in the 2nd episode	On the 81st day, she was discharged without sequelae in the 1st onset; relief of most symptoms in the 2nd onset	A mild gait disturbance at discharge. Resolution of most lesions at 2 months after discharge and symptomatic relief	Consciousness resumed on the 3rd day of the treatment. The patient was discharged with full recovery
Actual diagnosis and the reasons	Overlapping MOG-AD and anti-NMDARe existed in the 1st onset, MOG-AD existed in the 2nd onset.Reasons: Simultaneously meeting the diagnostic criteria of anti-NMDARe and MOG-AD, and involvement of opticnerves in the 1st onset	Anti-NMDARe existed in the 1st to the 3rd onset; MOG-AD existed in the 4th onset.Reasons: Coexistence of encephalopathy and no MOG-Ab in the 1st to the 3rd onset; optic neuritis; and good response to immunotherapy existed in the 4th onset	Anti-NMDARe existed in the 1st episode, and anti-NMDARe reappeared 3.5 years later.Reasons: She met the diagnostic criterion^[6]^ of anti-NMDARe;she did not have a good response toimmunotherapy; vision was unaffected	The more likely diagnosis was anti-NMDARe.Reasons: Encephalopathy existed; the patient had a poor response to multiple rounds of immunotherapy; vision is unaffected	The more likely diagnosiswas MOG-AD involving the hemicerebrum.Reasons: Good responseto immunotherapy. It did not meet the diagnostic criterion^[6]^ of typical anti-NMDARe

Most studies^[9,10,12–14]^ showed mildly elevated or normal leukocyte and protein levels in the CSF examination, consistent with our patient's findings. MRI in our case revealed that the lesions were located in the medial region of the superior frontal gyrus and involved the cingulate gyrus. Enhanced T1-weighted imaging showed enhancement in parts of the lesions. Interestingly, the shape and position of the lesions in reported cases^[5,12]^ were similar to that of our patient.

Unique features of MOG-AD are sensitivity to steroid use and withdrawal.^[7]^ The patient showed good response to a small amount of steroids, consistent with the diagnosis of MOG-AD.^[14]^ During follow-up (a minimum of 6 months), 58 (52.7%) patients with MOG-optic neuritis (MOG-ON) experienced at least one episode of recurrence of ON.^[17]^ Age of onset may be a potential predictor for determining visual prognosis of MOG-ON.^[17]^ Long-term outcome of patients with MOG-AD suggests infrequent relapse. However, some patients have persistently elevated MOG-Ab levels. These patients should have long-term follow-up due to the possibility of a relapse.^[18]^ Treatment plans must be individualized for the clinical features of MOG-AD or anti-NMDARe. Although there may be clinical improvement, long-term follow-up, including immunotherapy, may be required.

A complex relationship exists between anti-NMDARe and MOG-Ab; its definitive nature remains unknown. Further study of their co-occurrence and pathogenesis may deepen our understanding of this clinical entity.

In summary, the co-occurrence of MOG-Ab and anti-NMDAR-Ab in the test does not indicate the simultaneous presence of MOG-AD and anti-NMDARe. The positivity for these antibodies should be combined with the clinical features to achieve an accurate and suitable diagnosis. Accurate diagnosis plays an important role in treatment decisions and judgment of prognosis. Comprehensive screening for autoimmune antibodies is recommended for atypical cases of autoimmune diseases.

## Author contributions

**Conceptualization:** Liming Cao.

**Formal analysis:** Xuming Huang.

**Writing – original draft:** Liming Cao.

**Writing – review & editing:** Lijie Ren.
